# Comparing human to electronic observers to monitor hand hygiene compliance in an intensive care unit

**DOI:** 10.1017/ash.2022.303

**Published:** 2022-09-29

**Authors:** Eduardo Casaroto, Jose R. Generoso, Ary Serpa Neto, Marcelo Prado, Guilherme M. Gagliardi, Fernando Gatti de Menezes, Priscila Gonçalves, Fábio Barlem Hohmann, Guilherme Benfatti Olivato, Gustavo Potratz Gonçalves, Nathalia Xavier, Marcelo Fernandes Miguel, Michael B. Edmond, Alexandre R. Marra

**Affiliations:** 1 Hospital Israelita Albert Einstein, São Paulo, São Paulo, Brazil; 2 Australian and New Zealand Intensive Care Research Centre (ANZIC-RC), Monash University, ANZIC-RC, Melbourne, Victoria, Australia; 3 Universidade de São Paulo, São Carlos, São Paulo, Brazil; 4 West Virginia University School of Medicine, Morgantown, West Virginia, United States; 5 Center for Access & Delivery Research & Evaluation (CADRE), Iowa City Veterans’ Affairs Health Care System, Iowa City, Iowa, United States; 6 Department of Internal Medicine, University of Iowa Carver College of Medicine, Iowa City, Iowa, United States

## Abstract

**Objective:**

We sought to determine whether an electronic hand hygiene (HH) system could monitor HH compliance at similar rates to direct human observation.

**Methods:**

This 4-year proof-of-concept study was conducted in an intensive care unit (ICU) of a private tertiary-care hospital in São Paulo, Brazil, where electronic HH systems were installed in 2 rooms. HH compliance was reported respectively using direct observation and electronic counter devices with an infrared system for detecting HH opportunities.

**Results:**

In phase 1, HH compliance by human observers was 56.3% (564 of 1,001 opportunities), while HH compliance detected by the electronic observer was 51.0% (515 of 1,010 opportunities). In phase 2, human observers registered 484 HH opportunities with a HH compliance rate of 64.7% (313 of 484) versus 70.6% (346 of 490) simultaneously detected by the electronic system. In addition, an enhanced HH electronic system monitored activity 24 hours per day and HH compliance without the presence of a human observer was 40.3% (10,642 of 26,421 opportunities), providing evidence for the Hawthorne effect.

**Conclusions:**

The electronic HH monitoring system had good correlation with human HH observation, but compliance was remarkably lower when human observers were not present due to the Hawthorne effect (25%–30% absolute difference). Electronic monitoring systems can replace direct observation and can markedly reduce the Hawthorne effect.

Hand hygiene (HH) is a major infection control prevention strategy^1^ that reduces the transmission of pathogens between healthcare workers (HCWs) and patients.^2^

Direct observation is considered the gold standard method for evaluating HH compliance.^1,3,4^ However, the HH events (HHEs) observed represent a tiny fraction of the estimated number of HH opportunities, documented at 1.3% in one study.^5^ Studies that employ direct observation of HH are likely to be biased by the Hawthorne effect (ie, the presence of an observer positively influences HH compliance).^6^ This bias was demonstrated in a Joint Commission–led quality-improvement study in 2009 in which hospitals reported compliance rates of 85% via direct observation versus 48% measured using more accurate methods.^7^

Electronic HH counters on alcohol-based hand-rub (ABHR) dispensers are an important tool for obtaining HH data; they offer an automated method for capturing HH compliance in the hospital setting,^8–11^ particularly in the intensive care unit (ICU).^5^ Current evidence supports electronic HH-counter devices as a supplementary method, but it has not supplanted direct observation because current systems are not able to evaluate the quality of ABHR use and do not provide immediate peer-to-peer accountability.^5,10,12^

In this study, we sought to determine whether an electronic HH system (ie, an electronic HH observer) could monitor compliance at rates similar to those measured by direct human HH observation but in a more efficient way that would collect more data while mitigating the Hawthorne effect.

## Methods

This study was conducted in the ICU of a private tertiary-care hospital in São Paulo, Brazil. The ICU is a 40-bed, all private-room, medical-surgical unit with open staffing. The study was approved by the Hospital Israelita Albert Einstein Ethics Committee (CAAE 36179314.9.0000.0071). Informed consent was waived.

In this proof-of-concept study, we compared an electronic HH system to a human HH observer to monitor HH compliance over 4 years from October 1, 2016, to December 30, 2020. We installed electronic HH observers in 2 ICU rooms (A and B), which consisted of electronic counter devices with an infrared system to detect HH opportunities (Infectrack, i-HealthSys). This area has 4 hand-washing sinks, 1 per room and 2 outside the rooms. Care is provided by 1 nurse, 1 physician, 1 respiratory therapist, and 2 technicians.

For this study, 2 ICU physicians were trained by an infection preventionist (IP) in HH observation. The agreement of HH observations between the physicians and the IP was established in an ICU that was not part of the study. The ICU physicians and the IP observed HH performance in the same unit at the same time and compared their measured rates of compliance.^13^ The ICU physicians (not on clinical duty) were then directed to perform HH observations in the ICU rooms selected for the study for varying periods (ie, morning, afternoon, and night), randomly. The median duration of observation was 15 minutes. Data recorded on a mobile phone application were used to validate the HH opportunities performed by HCWs and captured by the electronic system.

If the ICU physicians (not on clinical duty but dressed in uniform) were questioned by HCWs, they would explain that they were observing problems that needed correction in the unit. As far as these physicians were aware, the ICU team never learned about the HH audits.

The HH events were registered by electronic handwash-counter devices with PURELL Instant Hand Sanitizer (70% ethyl alcohol + 4% isopropyl alcohol, 1-L bag; Gojo, Akron, OH). The hand-rub dispenser (NXT 1-L model) registers only 1 event every 2 seconds, even if >1 aliquot is dispensed. Chlorhexidine dispensers (chlorhexidine 2%) were also available for use, but these dispensers did not have electronic counters. Both dispensers released the same volume of product per use (∼1.3 mL) and were located inside patient rooms. All dispensers were the same type (ie, 1 L). Dispensers were checked and refilled as needed every 48 hours in both ICU rooms. Each room had 2 hand-rub dispensers, and a shared dispenser was located outside these 2 rooms.

**Fig. 1.**
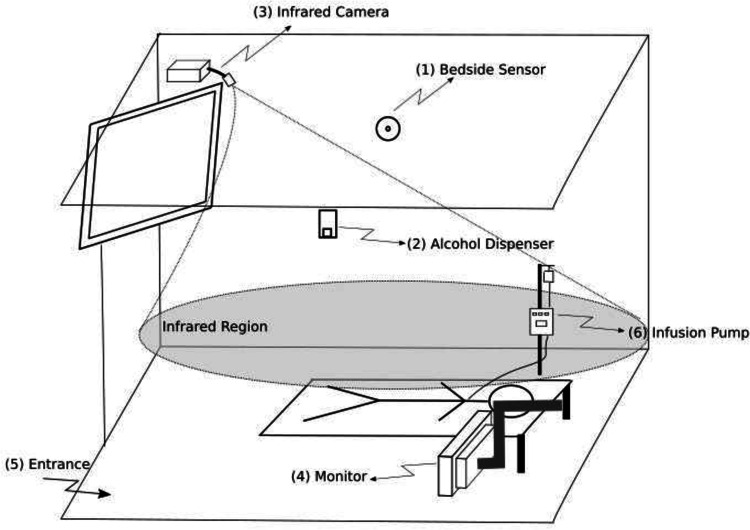
Infrared system installed inside the room: (1) bedside sensor, (2) alcohol-based handrub dispenser, (3) infrared camera, (4) monitor, (5) room entrance and exit, and (6) infusion pump. The grey area represents the area captured by the infrared camera.

**Fig. 2.**
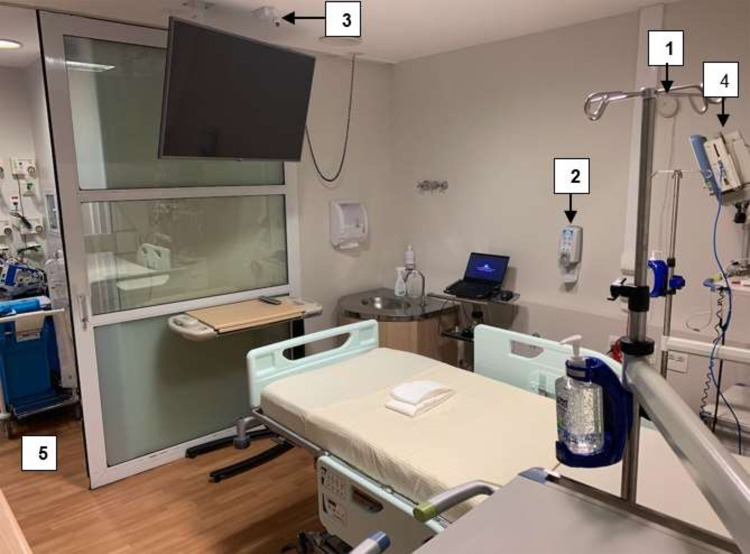
Room photo showing some items depicted in Figure 1: (1) bedside sensor, (2) alcohol-based handrub dispenser, (3) infrared camera, (4) monitor, (5) room entrance and exit.

In the 2 ICU rooms, sensors were used to obtain infrared images of HH opportunities between the HCWs, the patient, and the environment (eg, ICU equipment including infusion pumps, monitors, and counters where medicines are prepared). The system was comprised of (1) a bedside HH sensor, (2) alcohol dispensers with badge-detection sensors, (3) a camera with an infrared sensor installed on the ceiling, (4) a multiparameter monitor, (5) room entrance and exit, and (6) an infusion pump (Fig. 1 and Fig. 2).

Feedback loop or real-time feedback methodology uses a wireless identification device for the HCW who performs HH with alcohol hand rub via the dispenser inside the patient room. The identification devices, which look like an ID badge, use Zigbee technology wireless communication protocol (based on IEEE 802.15.3 standards).

In addition, a warning sensor located on the bed wall triggered a warning light via wireless technology when an HCW approached the bed. A red light flashed above the patient’s bed when an HCW approached and hand hygiene was not performed; a green light flashed if HH was performed. Software integrated with a database reported how many HCWs entered the rooms, how many performed HH, and how many patients were provided with care. However, the HCWs were only recognized by this system if they wore the identification device with Zigbee technology. Identification devices were worn by nurses, nursing technicians, physical therapists, and physicians.

The ICU rooms were equipped with other hand-sanitizer dispensers apart from the ones used for the project, positioned at strategic points inside the room. There was no intervention for HCWs to use nonelectronic hand sanitizers. We did not have hand hygiene electronic counters to monitor whether HCWs washed their hands with water and soap. However, from direct observation we know that, in our ICU, >90% of the hand hygiene product used was alcohol gel.^14^

Both devices, the feedback loop and the infrared system, consisted of small devices. The first was located at the bed wall and the second one was located above the television. In most cases, the patient did not perceive their presence. The system was designed to work discretely to preserve the patient’s privacy.

After the installation of the HH and infrared system inside the ICU rooms and calibration of the system based on human observation performed by the physicians from October 2016 to August 2018, the project was divided into 2 phases. In phase 1, data collected by the human observers were compared to electronic monitoring data from the feedback loop and infrared system only during the periods of human observation (September 2018 to June 2020). In phase 2, the human observers collected data up to 15 minutes daily, whereas the infrared system monitored 24 hours per day, generating a larger amount of data (July 2020 to December 2020). Electronic data were compared to data obtained by human observers contemporaneously. The electronic system also monitored compliance when the human observer was no longer present, representing an enhanced electronic observation.

**Fig. 3.**
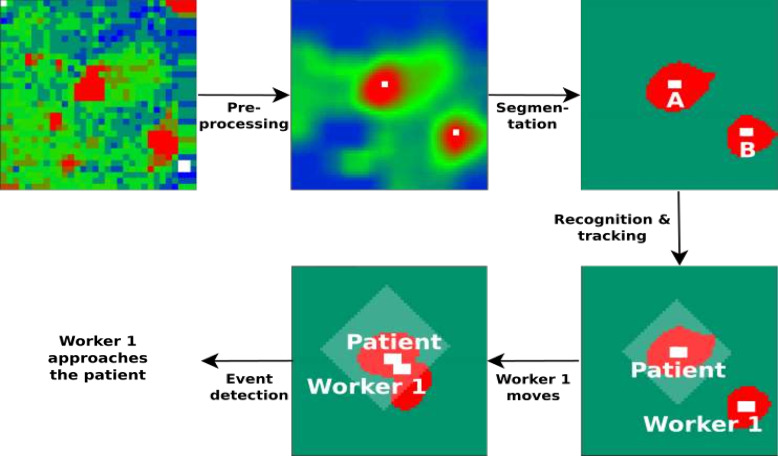
Process used to identify physical contact events using machine learning image segmentation (phase 2).

It illustrates how an image is processed using a machine learning algorithm (Fig. 3). The algorithm performs a preprocessing analysis of the image so that it is possible to segment the image in a sequence. After segmentation, when the position of the patient’s bed is known, the algorithm detects the patient and the HCW. The algorithm detects and records physical contact events between the HCW and the patient. All detected physical contact events are processed together with data from the HH electronic system, and compliance rates related to HH opportunities are calculated according to Your Five Moments for HH, as recommended by the World Health Organization (WHO)^15^. The same algorithm detects physical contact events between the HCW and the equipment inside the room.

### Statistical analysis

Qualitative data were described by absolute frequencies and percentages and were compared using the χ^2^ test. HH compliance was calculated by dividing the number of HHE performed when an opportunity existed by the total number of HH opportunities.^15^ We calculated accuracy using the following formula: 100% (observed value − reference value)/reference value ×100, where the observed value is the electronic observer, and the reference value is the human observer. Analyses were conducted using R program software (R Foundation for Statistical Computing, Vienna, Austria).

## Results

The agreement between the physicians with the IP for 189 HH opportunities yielded a κ (kappa) coefficient of 0.97 (95% CI, 0.93–1.01; *P* < .001). Table 1 presents HH human observations in both phases 1 and 2.

**Table 1.** Data Collected by Human Observers

Variable	Phase 1, No. of Events (Compliance Rate, %)	Phase 2, No. of Events (Compliance Rate, %)
Weekend	963 (96.2)	468 (96.7)
**Time of day**		
Morning (07:00–12:59)	334 (33.4)	308 (63.6)
Afternoon (13:00–18:59)	374 (37.4)	152 (31.4)
Night (19:00–06:59)	293 (29.3)	24 (5.0)
**Hand hygiene opportunity**		
Before touching a patient	363 (36.3)	168 (34.7)
Before clean or aseptic procedures	30 (3.0)	1 (0.2)
After body fluid exposure or risk	35 (3.5)	10 (2.1)
After touching a patient	222 (22.2)	114 (23.6)
After touching patient surroundings	351 (35.1)	191 (39.5)
Hand-rub hygiene performed	564 (56.3)	313 (64.7)

Table 2 summarizes the number of HHEs registered by human and electronic observers and the HH compliance in each phase. In phase 1, human observers registered 564 HHEs, demonstrating an HH compliance rate of 56.3% (564 HHEs of 1,001 HH opportunities), whereas the electronic observer registered 132,953 HHEs, demonstrating an HH compliance rate of 51% (515 HHEs of 1,010 HH opportunities; *P* = .016).

**Table 2.** Summary of the Number of Hand Hygiene Events (HHEs) and Compliance in Each Study Phase

Variable	Phase 1^a^	Phase 2^b^
**Hand hygiene events, no. (%)**		
Human observer	564 (0.4)	313 (1.1)
Electronic observer	132,953 (99.6)	27,047 (98.9)
**Hand hygiene compliance, %**		
Human observer	56.3	64.7
Electronic observer with human observer present	51.0	70.6
Enhanced electronic observer with no human observer present	NA	40.3

Note. HHEs, hand hygiene events; NA, not available.

a In phase 1, comparing human observer versus electronic observer with human observer present (*P* = .016).

b In phase 2, comparing human observer versus electronic observer with human observer present (*P* = .048).

In phase 2, human observers registered 313 HHEs, demonstrating an HH compliance rate of 64.7% (313 HHEs of 484 HH opportunities), whereas the electronic observer registered 27,047 HHEs, demonstrating an HH compliance rate of 70.6% for the electronic observer (346 HHEs of 490 HH opportunities) using data obtained only within the presence of a human observer (accuracy, 81%; *P* = .048). Also in phase 2, the enhanced electronic monitoring (24-hour electronic system with infrared cameras in the absence of a human observer) registered a 40.3% HH compliance rate (10,642 HHEs of 26,421 HH opportunities), which was 24.4% lower than that measured by human observers (64.7%). This difference is explained by the Hawthorne effect. The electronic system also monitored 24 hours a day, showing that HH compliance was significantly lower than that measured by human observers.

## Discussion

In this study, we compared HH rates detected by electronic systems and human observers. The capture rates of HH events were similar, but the compliance rates were quite different due to the Hawthorne effect. Our enhanced electronic HH system was designed to ensure that HCWs perform HH prior to patient care, issuing an automatic warning to do so.^16^ With the enhanced electronic observer monitoring continuously, 24 hours a day, the number of opportunities observed was very high, reaching >26,000 opportunities captured by the infrared camera in 6 months.

Electronic HH systems are an important tool for obtaining data about HH, offering a novel and automatic way of registering HH adherence in the hospital setting, principally in the ICU. Current evidence supports electronic HH counter devices as a supplementary method, but it has not supplanted direct observation to date. Current systems are not able to evaluate the quality of HCW performance using alcohol hand rub, and they do not allow for immediate peer-to-peer correction. We did not perform any intervention in our study, we only compared human observers with electronic observers in 2 different phases (basic and enhanced electronic HH system) to validate the electronic HH system for 24 hours.

The Hawthorne effect has a “performance ceiling.”^17^ In one study, the influence of the Hawthorne effect in HH increased over time until this performance ceiling was reached.^18^ The compliance rate increased in the first 10 minutes of observation but started to flatten at 95%, as auditors stayed >15 minutes in the ward. Limiting direct observation periods to ∼15 minutes, to minimize the Hawthorne effect and to determine the required number of hand hygiene opportunities observed per period on the basis of statistical power calculations, are expected to improve the validity of HH surveillance programs.^19^ The Hawthorne effect has been demonstrated to introduce a significant amount of bias when HH compliance is measured via overt observation,^20^ a method almost universally used by hospitals.^4,19,21–23^ In a study by Scherer et al, the absolute difference in HH compliance estimated between the standard and the new 15-minute audit method (secret shopper) was ∼30%.^20^ Generally, hand hygiene studies using human observers include 60-minute observation periods,^3,6^ whereas electronic counters can record 24 hours per day.

We developed an electronic HH system that recorded every HH opportunity and every aliquot of hand rub dispensed. An infrared system capable of detecting physical contact opportunities between HCWs and patients and the environment was developed. The algorithm developed for an infrared system was based on image segmentation using machine-learning techniques (Fig. 3). It was possible to generate an HH compliance percentage rate because the enhanced HH monitoring system utilized an infrared system. This coupled system increased the precision of HH compliance rates using improved discrimination of HH opportunities (eg, when a HCW entered the room and did not touch the patient or the environment, no HH opportunity was recognized).

A long study period was necessary to validate the technological methods. Development of the hardware (radiofrequency sensors, badges, infrared camera and local server with WiFi connection) and software (ie, a mobile application to collect initial data and the algorithm created via machine learning to translate the images captured through infrared camera into data) took several months.

Much work remains in standardized direct-observation practices, particularly training and validating observers if >1 observer will be collecting data.^13^ Reliability among observers is often referred to as observer interrater reliability or observer interrater agreement. We employed just 2 observers to perform HH observation while an electronic hand hygiene system was undergoing validation.

Since WHO published its guidelines, the definition of HH opportunities has been focused on the Five Moments for Hand Hygiene: before patient contact (moment 1), before aseptic tasks (moment 2), after body fluid exposure risk (moment 3), after patient contact (moment 4), and after contact with patient surroundings (moment 5).^15,24^ In our study, moments 1, 4, and 5 represented almost 80% of all HH opportunities in both phases, which is comparable to the findings of other studies.^10,25^ We believe that moments 2 and 3 were difficult to observe due to the patient privacy arrangements and the architectural features of the ICU.^26–28^ However, part of our study was performed during the COVID-19 pandemic; it was challenging to employ observers during this unusually critical time in our ICU.^29,30^

Another standard method is to monitor HH at entries and exits. Because most US facilities have wall-mounted hand-rub stations outside patient rooms due to fire codes, it is significantly easier to observe entry–exit compliance with HH practices compared to the WHO HH guidelines.^28^ However, many opportunities inside the patient room are missed. We are unaware of any electronic HH system able to observe as many HH opportunities as a human observer. In our study, however, we combined enhanced HH sensors (RFID and infrared sensor) in an ICU room to collect data on HH opportunities, thus offering an HH compliance rate.

Our study had several limitations. If an HCW was not wearing their badge, the system could not assess which room the HCW had entered. The recording of HHEs was independent of HCW use of badges. The badges only give a reminder (feedback loop) for HCWs to remember to perform hand hygiene. Additionally, we encountered technical issues with the enhanced electronic HH system almost 50% of the time during the validation phase, including replacement of the motherboard, HCWs changing the position of the infrared camera, and delays in software installation. Another technical issue we encountered was that the infrared camera was not running continuously until September 2020. Due to the high cost of infrared cameras, we were also unable to add them to more ICU rooms. And finally, we were not able to demonstrate that HCWs always used their badges while on duty. We believe that HCWs considered this inconvenient and may have been concerned about being monitored during their work.^31^

This study also had several strengths. We demonstrated that establishing an electronic feedback loop is possible. The sensors and infrared cameras were small, discrete, and silent, and they did not interfere with the patient’s privacy or bother the patient. This system does require adaptation in the patient’s room, and once validated, it is easy to install and calibrate. Infrared monitoring has been patented in the United States.^32^

This study is not conclusive, and its scope could have been greater. The integration of these new technologies with medical equipment or within inpatient beds is not yet widespread, likely due to cost and difficulty of implementing the necessary infrastructure. Even institutions with the financial capacity to implement HH electronic systems need to consider an interface with engineering specialists to determine whether they may interfere with existing equipment or overload existing wireless networks.^10^ Electronic HH monitoring systems are promising, but more research is needed for adequate validation.^33–37^

In conclusion, the electronic HH system (electronic observer) used in this study had good correlation with the direct observation method (human observer), producing similar HH compliance rates with good accuracy. However, this electronic system has the potential to produce more realistic HH compliance rates by minimizing the Hawthorne effect.
